# Transcriptome and proteome analysis of innate immune responses to inactivated *Leptospira* and bivalent *Leptospira* vaccines in canine 030-D cells

**DOI:** 10.1038/s41598-022-16457-z

**Published:** 2022-08-04

**Authors:** Andreja Novak, Jeroen L. A. Pennings, Larissa van der Maas, Hugo D. Meiring, Irene Ludwig, Saertje Verkoeijen, Victor Rutten, Femke Broere, Arjen Sloots

**Affiliations:** 1 Division of Infectious Diseases and Immunology, Department of Biomolecular Health Sciences, Faculty of Veterinary Medicine, Utrecht University, Utrecht, The Netherlands; 2 Intravacc, Bilthoven, The Netherlands; 3 Centre for Health Protection, National Institute for Public Health and the Environment (RIVM), Bilthoven, The Netherlands; 4 Research Centre Healthy and Sustainable Living, Innovative Testing in Life Sciences and Chemistry, University of Applied Sciences Utrecht, Utrecht, The Netherlands; 5 Department of Veterinary Tropical Diseases, Faculty of Veterinary Science, University of Pretoria, Pretoria, South Africa; 6 Division of Internal Medicine of Companion Animals, Department of Clinical Science, Faculty of Veterinary Medicine, Utrecht University, Utrecht, The Netherlands

**Keywords:** Inactivated vaccines, Bacterial infection, Monocytes and macrophages, Innate immunity

## Abstract

Mandatory potency testing of *Leptospira* vaccine batches relies partially on in vivo procedures, requiring large numbers of laboratory animals. Cell-based assays could replace in vivo tests for vaccine quality control if biomarkers indicative of *Leptospira* vaccine potency are identified. We investigated innate immune responsiveness induced by inactivated *L. interrogans* serogroups Canicola and Icterohaemorrhagiae, and two bivalent, non-adjuvanted canine *Leptospira* vaccines containing the same serogroups. First, the transcriptome and proteome analysis of a canine monocyte/macrophage 030-D cell line stimulated with *Leptospira* strains, and vaccine B revealed more than 900 DEGs and 23 DEPs in common to these three stimuli. Second, comparison of responses induced by vaccine B and vaccine D revealed a large overlap in DEGs and DEPs as well, suggesting potential to identify biomarkers indicative of *Leptospira* vaccine quality. Because not many common DEPs were identified, we selected seven molecules from the identified DEGs, associated with pathways related to innate immunity, of which CXCL-10, IL-1β, SAA, and complement C3 showed increased secretion upon stimulation with both *Leptospira* vaccines. These molecules could be interesting targets for development of biomarker-based assays for *Leptospira* vaccine quality control in the future. Additionally, this study contributes to the understanding of the mechanisms by which *Leptospira* vaccines induce innate immune responses in the dog.

## Introduction

Leptospirosis is a global zoonotic disease caused by invasive Gram-negative *Leptospira* (*L.)*. Based on the carbohydrate structure of their lipopolysaccharides (LPS), more than 200 serovars have been identified of which many are of unknown clinical relevance^1^. According to literature, only 6–8 serogroups are known to cause disease in dogs^2^. Because of the zoonotic potential and severity of disease, vaccination of cattle, pigs and dogs is widely used to protect both humans and animals against leptospirosis^3^. Commercially available canine *Leptospira* vaccines are bi- or multivalent and consist of chemically or physically inactivated whole bacterial cell vaccines (bacterins). *Leptospira* vaccines are serovar specific and annual revaccination is required to maintain immunity^4^. Bivalent vaccines containing whole-inactivated *L. interrogans* serogroups Icterohaemorrhagiae and Canicola^5,6^ have been on the market since the 1960s^4^. More recently, multivalent canine *Leptospira* vaccines additionally containing inactivated serogroups Grippotyphosa and Australis were developed to protect against a broader range of *Leptospira* serogroups^7^.

Potency testing of human and veterinary vaccine batches is mandatory prior to release to the market to ensure that the quality of each batch meets the regulatory requirements^3^. Potency tests of non-replicating vaccines traditionally assess the immune response to the vaccine by serology or challenge tests in target or laboratory animals^8^. Concerns for animal welfare while performing animal-based potency assays, as well as costs, duration and poor reproducibility of these quality control assays, stimulated implementation of the 3R’s concept (reduction, refinement, and replacement) in regulatory testing of vaccines^9,10^. Furthermore, the implementation of product monitoring systems such as good manufacturing practice (GMP) and quality assurance (QA), as well as process standardization improved consistency in the traditional vaccine batch production pipeline. Together, these developments allow replacement of traditional animal-based potency assays of inactivated vaccines by cell-based in vitro potency tests for batch consistency^9,11^. Recently, several studies focused on identifying biomarkers indicative of consistent vaccine batch production^12–14^ or vaccine-induced immune responses^15–17^, in attempts to develop non-animal based potency tests for vaccine quality control.

Potency testing of canine *Leptospira* vaccines relies on a Golden Syrian hamster challenge test where vaccinated and non-vaccinated animals are challenged with a live *Leptospira* strain. Because of the large number of laboratory animals used and the pain and distress inflicted on the animals by this approach^3,18,19^, serovar specific monoclonal antibodies, recognising LPS epitopes were used in an enzyme-linked immunosorbent assay (ELISA) to quantify *Leptospira* antigen present in bacterins, as a potency assay^20–23^. ELISAs were validated for the quantification of serovars Canicola and Icterohaemorrhagiae present in non-adjuvanted canine *Leptospira* vaccines^24^, comply with regulatory requirements^25^ and are currently widely used by *Leptospira* vaccine manufacturers. Although the LPS-based ELISA is a reproducible and affordable alternative to the hamster potency assay^20–24^, it quantifies a single epitope per individual serovar of the vaccine and does not provide information about antigen processing or immune activation by the vaccine^26–28^. In addition, all vaccine components must be tested separately, diminishing the cost-advantage of an ELISA-based in vitro assay. Therefore, a cell-based assay assessing the functional immunostimulatory capacity of a vaccine could be a valuable tool, additional to the ELISA, for quality control of *Leptospira* vaccine batches in vitro*.*

The innate immune system is part of the early host defence against invading pathogens. Upon vaccination or infection, microbe-associated molecular patterns (MAMPs) present in the vaccine or the pathogen, trigger host innate immune responses through pattern recognition receptors (PRRs) expressed on several cell types including innate immune cells such as neutrophils, NK cells, monocytes, macrophages and dendritic cells^29^. In previous studies the canine 030-D cell line^30^ was proposed as a model to study innate immune responses in dogs in vitro^31,32^. Furthermore, a cell line is a stable alternative for the development of an in vitro assay. In the present study, we stimulated the 030-D cell line with inactivated bacteria of *L. interrogans* serogroups Canicola and Icterohaemorrhagiae, and two non-adjuvanted bivalent canine *Leptospira* vaccines containing the same inactivated *Leptospira* serogroups to investigate *Leptospira* vaccine-induced responses on the transcriptome and proteome level using RNA sequencing and mass spectrometry. Firstly, our findings contribute to the understanding of molecular mechanisms by which non-adjuvanted canine *Leptospira* vaccines induce innate immune responses in the dog. Secondly, this study identified several molecules, including CXCL-10, IL-1β, SAA, and complement factor C3, that could serve as markers for *Leptospira* vaccine quality and, therefore, may be interesting targets for the development of a biomarker-based in vitro assay for quality assessment of multivalent canine *Leptospira* vaccines.

## Materials and methods

### *Leptospira* strains and vaccines

Inactivated *L. interrogans* serogroup Canicola serovar Portland-vere (strain Ca-12-000) and *L. interrogans* serogroup Icterohaemorrhagiae serovar Copenhageni (strain Ic-02-001), bivalent *Leptospira* vaccine containing these two *Leptospira* strains (vaccine B), and excipient containing all vaccine components except the antigen were kindly provided by a pharmaceutical company that is part of the VAC2VAC consortium (http://www.vac2vac.eu/), hereafter referred to as company B. A second bivalent *Leptospira* vaccine containing inactivated *L. interrogans* serogroup and serovar Canicola strain 16,070 and *L. interrogans* serogroup and serovar Icterohaemorrhagiae strain 16,069 (vaccine D) was kindly provided by another pharmaceutical company also participating in the VAC2VAC consortium, hereafter referred to as company D.

### Stimulation of 030-D cells with inactivated *Leptospira* strains and vaccines

The canine 030-D cell line, derived from a canine malignant histiocytosis^30^, was cultured at 37 °C and 5% CO_2_ in culture medium consisting of RPMI-1640 (Gibco) supplemented with 10% heat-inactivated fetal bovine serum (FBS; Gibco), 100 U/ml penicillin, 100 µg/ml streptomycin and 300 ng/ml L-glutamine (all Gibco). For stimulation assays, 4 × 10^6^ 030-D cells were seeded in 25 cm^2^ flasks (Greiner) in 5 ml culture medium and incubated over night at 37 °C and 5% CO_2_. Cells were stimulated in 12 replicates with predetermined highest dose (or lowest dilution) that did not induce toxic effects, but still induced cytokine expression of the inactivated whole-cell preparations of *L. interrogans* serogroup Canicola or Icterohaemorrhagiae (1:10 dilution), excipient (1:200 dilution) as non-antigen control and bivalent *Leptospira* vaccine (1:200 dilution) (all company B). Furthermore, the 030-D cell line was stimulated with an additional bivalent *Leptospira* vaccine, produced by company D (1:50 dilution). Cells were incubated for 8 or 24 h at 37 °C and 5% CO_2_ and harvested for transcriptome or proteome analyses in three replicates per time-point. In addition, unstimulated control (medium only) 030-D cells were harvested after 0, 8 and 24 h incubation.

### RNA isolation

Cells were harvested from three replicates for each condition at 8 and 24 h stimulation and centrifuged 5 min at 300×*g*. The pellet was suspended in 200 µl PBS and lysed in 550 µl Lysis/binding buffer (Roche) supplemented with 1% DTT (Sigma Aldrich). Cell lysates were stored at − 80 °C until RNA isolation with the MagNA Pure LC RNA isolation kit-High performance (Roche) on the MagNA Pure LC 2.0 instrument following the manufacturer’s protocol. Isolated RNA samples were quantified using UV spectroscopy (Take3 module, Synergy Mx, BioTek, Winooski, USA). RNA integrity was analysed by agarose gel electrophoresis using 2% E-Gel EX pre-stained gels (Thermo Fischer Scientific).

### RNA sequencing and data analysis

For library preparation, total RNA and ERCC RNA Spike-In Mix 1 (Thermo Fisher Scientific) were combined. Poly-A enrichment was done with the NEBNext Poly(A) mRNA Magnetic Isolation Module (New England BioLabs). RNA-Seq libraries were generated using the NEBNext Ultra II Directional RNA Library Prep Kit and NEBNext Multiplex Oligos for Illumina (Unique Dual Index Primer Pairs) (New England BioLabs) following manufacturers’ protocols. The size distribution of the libraries with indexed adapters was assessed with Agilent D1000 ScreenTapes on a 2200 TapeStation System (Agilent Technologies). The libraries were quantified using the NEBNext Library Quant Kit for Illumina (New England BioLabs) on a QuantStudio 3 Real-Time PCR System (Thermo Fisher Scientific) following manufacturers’ protocols. The 75 bp libraries were sequenced using a NextSeq 500/550 High Output Kit (75 Cycles) on a NextSeq 550 Sequencing System (Illumina) at MAD: Dutch Genomics Service & Support (University of Amsterdam, The Netherlands). The first read of the paired-end reads were used for data analysis. These FASTQ files were treated as single reads by our RNA-Seq pipeline. Raw FASTQ files were then subjected to a quality control procedure using FastQC (version 0.11.5) (www.bioinformatics.babraham.ac.uk/projects/), and checked for any biases and outliers such as library size, read length distribution, mean read quality distribution and mean quality for each position in the read as well as base frequency for each position in the read. Next, FASTQ reads were mapped to the dog genome (genome assembly: CanFam3.1, release 97) using STAR (version 2.5.3a)^33^. The number of mapped reads were counted for each gene and compiled into an expression matrix using featureCounts (version 1.6.0)^34^. This resulted in a table with gene counts for 32,705 genes. Complete raw fastq data, count data, and metadata from this publication are available at GEO (www.ncbi.nlm.nih.gov/geo/) under accession number GSE192945. Further statistical data analysis and visualization was done in R statistical software (version 3.6.0) (www.R-project.org)^35^. R packages used included DESeq2, limma, and gplots. Genes that had zero counts in all samples were considered unexpressed and discarded from further analysis. Gene count data were normalized using a variance stabilizing transformation (VST) on the remaining 21,448 genes. Differential gene expression was determined by means of a one-way ANOVA between different treatment conditions at 8 h and 24 h. Genes were considered to be significantly differentially expressed if the raw p-value was < 0.001, the adjusted p-value was < 0.05 (based on the Benjamini–Hochberg False Discovery Rate (FDR))^36^ and the absolute Fold Change (FC) compared to the unstimulated control was > 2. Genes with the absolute FC > 16 were considered highly expressed. Sets of differentially expressed genes (DEGs) were compared with a Venn diagram or scatter plot. Additional visualization of the data was done using a heatmap combined with hierarchical clustering (using Euclidean distance and Ward linkage). For a comparison of gene expression in the 030-D cell line to that in various human blood cell types, gene count data were converted to transcripts per million (TPM) values and analysed with the shiny app using the ABIS RNA seq tool (https://giannimonaco.shinyapps.io/ABIS/)^37^. Cellular functions and pathways over-represented in the DEG list were assessed using DAVID Bioinformatics Resources 6.8^38,39^ and KEGG database (https://www.kegg.jp/kegg/kegg1.html)^40^.

### Protein digestion, labelling and LC–MS/MS analysis

At each time point, cells were harvested from 3 replicates for each condition, centrifuged 5 min at 300*g* and resuspended in 100 µl 50 mM phosphate buffer (Sigma Aldrich). Proteins were denatured by adding RapiGest SF Surfactant (Waters Corporation, USA) to a final concentration of 0.1% (w/v) and incubation at 80 °C in a water bath for 30 min after which the samples were stored at − 80 °C until protein digestion. The protein content was determined by assessing UV absorbance at 280 nm on a Nanodrop spectrophotometer (Thermo Fisher Scientific) according to the manufacturer’s instruction. Protein digestions were performed on samples containing 200 µg/ml protein in 100 mM phosphate buffer pH 7.4 (Sigma Aldrich) with the addition of 2 µl of 0.1 mg/ml Lys-C (Roche) and incubation for 4 h at 37 °C, followed with an addition of 2 µl of 0.4 mg/ml trypsin (Promega) and overnight incubation at 37 °C. Dimethyl labelling was used for the quantitative analysis of protein expressions by mass spectrometry as previously described^15^ with minor modifications. Native formaldehyde (CH_2_O with M = 30.03 g/mol; Sigma Aldrich) and sodium cyanoborohydride (NaCNBH_3_; Sigma Aldrich) were used at final concentrations of 45 mM for “light” labeling of digested samples. The deuterated formaldehyde (CD_2_O with M = 32.04 g/mol; Sigma Aldrich) and NaCNBH_3_ were used at final concentrations of 45 mM for “heavy” labelling of the internal standard (“Common reference”). The labelling of samples and Common reference was performed at 37 °C for 2 h. Each “light”-labeled sample was mixed with an aliquot of the “heavy”-labeled Common reference, based on equal protein amounts for the “light” and “heavy”-labeled samples. The LC–MS/MS analysis was performed exactly as previously described^15^.

### Processing of the LC–MS/MS data

LC–MS data were processed with PEAKS X (Bioinformatics Solutions Inc., Canada) against the *Canis lupus familiaris* database (Taxonomy ID = 9615, www.uniprot.org) containing 45,351 entries for protein identification. Enzyme specificity was set to trypsin (semispecific) with a maximum of 3 missed cleavages. The parent mass error tolerance and fragment mass error tolerance for ions were set to 20 ppm and 0.6 Da, respectively. Static modifications were carbamidomethylation on Cys (+ 57.02 Da), dimethylation on Lys and the peptide N-termini (+ 28.0313 Da and + 32.0564 Da for "light" and "heavy", respectively), while variable modifications were deamidation on Asn and Gln (+ 0.98402 Da) and oxidation on Met (+ 15.99492 Da). False Discovery Rate (FDR) was set to 1%. Chromatographic peak area intensities of the precursor ions of the identified peptides were used for relative protein quantification. Complete raw data, processed data and metadata from this publication are available at PRIDE (http://www.ebi.ac.uk/pride) under accession number PXD031875.

Protein MS data processing and statistical analysis were done using R statistical software on the 2623 identified proteins (here defined as different UniProt accession numbers). The relative quantification of the samples was based on the comparison of all samples against a Common Reference acting as an internal standard between samples, in accordance with previous publications^15,41^. The resulting sample / Common Reference ratios were Log2-transformed. Next, the values were normalized for variations between measurements by performing a median correction, in which all relative protein expression values were divided by median protein expression value of each run. Proteins were included as quantitative differentially expressed proteins (DEPs) if (i) they were detected in at least two out of three replicates for both stimulated and unstimulated samples, (ii) showed an average upregulation or downregulation of at least twofold upon stimulation, and (iii) change was statistically significant (p < 0.01) according to a Student’s T-test. Additionally, proteins were considered as qualitative DEPs if they were detected in all samples of the stimulated group and in no samples of the unstimulated group, or the reverse situation. Quantitative and qualitative DEPs were compared between exposures using Venn diagrams. The subcellular localization of DEPs was determined based on GO terms for cellular component using QuickGO (https://www.ebi.ac.uk/QuickGO/) and UniProtKB databases.

### Cytokine analysis

For cytokine analysis, 1 × 10^6^/ml 030-D cells were stimulated for 24 h in a 96-well plate using the same dilutions of the inactivated *Leptospira*, vaccines and excipient as described in section “Stimulation of 030-D cells with inactivated *Leptospira* strains and vaccines”. Supernatants were stored at − 20 °C until analysis. ELISA kits for canine IL-1α, IL-1β, CXCL-10 and IL-8 (Kingfisher Biotech, Inc) were used in in-house developed multiplex cytokine assays using the Magpix system (Luminex XMAP) according to the manufacturer’s instructions. The cytokine concentrations in supernatants of stimulated cells were calculated using the standards provided in the kits. The MFI data were analysed using a 5-parameter logistic method (xPONENT software, Luminex, USA). In addition, canine S100A12 (Antibodies-online), SAA and complement factor C3 (C3) (both Abcam) ELISAs were performed according to manufacturer’s instructions.

### Statistical analysis

Expression of proteins in the supernatant of stimulated cells (Fig. 5) was analysed with One-way ANOVA and Dunnett’s multiple comparisons tests using GraphPad Prism 9 software (GraphPad Software Inc., San Diego, CA, USA). A p-value of < 0.05 was considered statistically significant.

## Results

### Inactivated *Leptospira* and bivalent *Leptospira* vaccine B induce similar responses of the canine 030-D cell line on the transcriptome level

We analysed the transcriptome of the canine 030-D cell line after 8 and 24 h of stimulation with inactivated *L. interrogans* serogroup Canicola or Icterohaemorrhagiae, bivalent *Leptospira* vaccine B containing these two inactivated *Leptospira* strains, or vaccine excipient as non-antigen control to capture early and late changes in gene and protein expression. Paired-end RNA-seq libraries were generated and mapped to the dog genome which resulted in the identification of 32,705 genes. After excluding unexpressed genes, 21,448 were included in analyses. First, to confirm the suitability of the 030-D cell line to study innate immune responses, we compared our transcriptome data set to the library of transcriptome profiles of human peripheral blood mononuclear cell (PBMC) subsets defined by Monaco et al*.* using the ABIS RNA-seq tool^37^. This analysis revealed that the phenotype of 030-D cells compared best to non-classical and intermediate monocytes, suggesting that the 030-D cell line is suitable to study innate immune responses in vitro (Supplementary Table S1).

**Figure 1:**
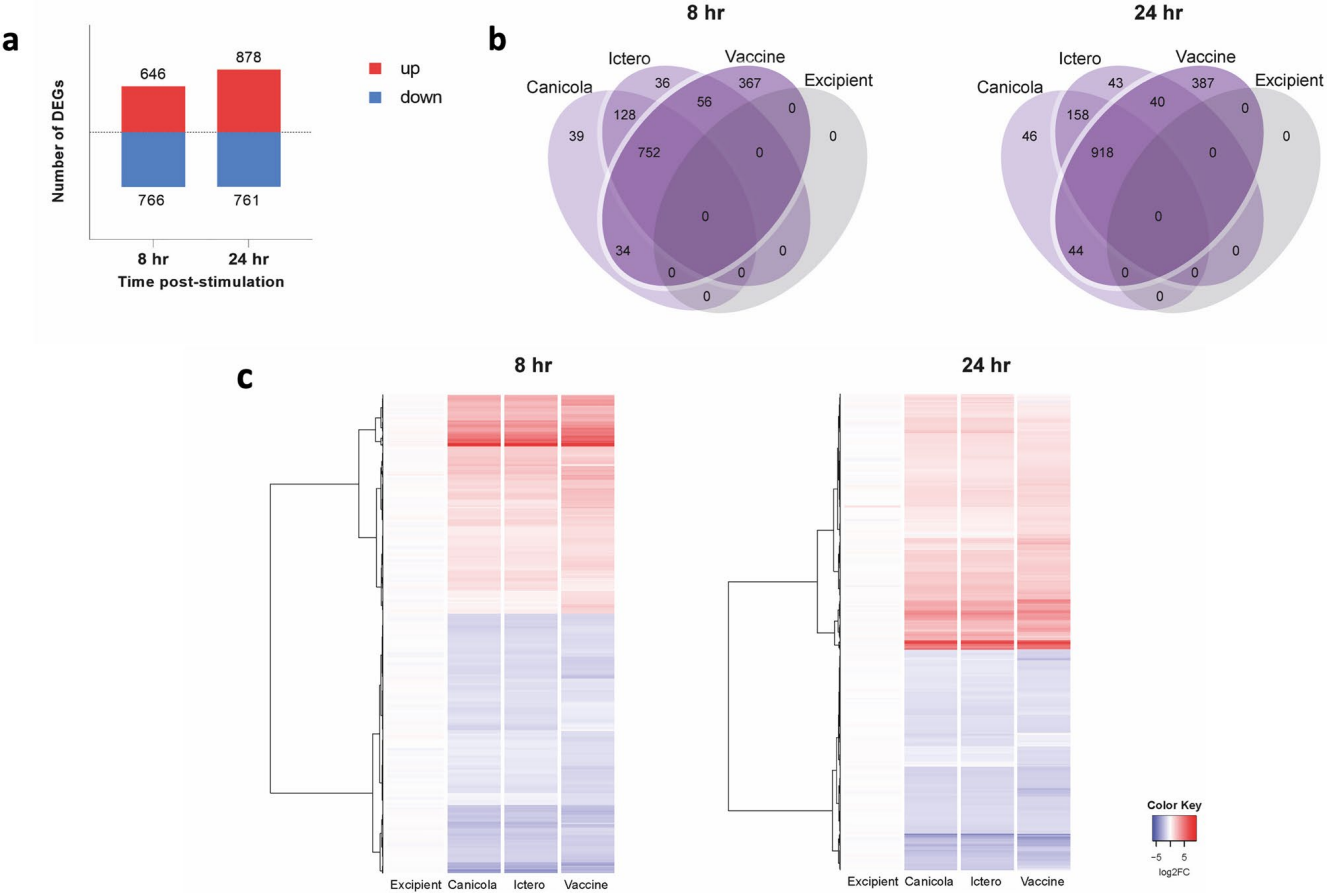
DEGs (FC ≥ 2; P < 0.001, FDR 5%) found in canine 030-D cells after 8 and 24 h stimulation with the inactivated *L. interrogans* serogroup Canicola or Icterohaemorrhagiae or bivalent *Leptospira* vaccine B. (**a**) The number (y-axis) and directionality (upregulated = positive y-space, downregulated = negative y-space) of DEGs after stimulation with the inactivated *L. interrogans* serogroup Canicola or Icterohaemorrhagiae, bivalent *Leptospira* vaccine containing the same *Leptospira* strains or vaccine excipient as non-antigen control (all manufacturer B). (**b**) Venn diagram of DEGs shown in (**a**). (**c**) Heatmap representing hierarchical clustering of DEGs for all stimuli. The color key represents the changes (log2 scale), from most downregulated (dark blue) to most upregulated genes (red). Each row is a gene, and each column is a stimulus (average of n = 3 replicates).

To identify potential marker genes, representative of canine *Leptospira* vaccine potency, we compared changes in the transcriptome of 030-D cells induced by inactivated *Leptospira* strains, a bivalent *Leptospira* vaccine B and vaccine excipient after 8 and 24 h stimulation (P < 0.001, FDR 5%, FC ≥ 2; Fig. 1a–c and Supplementary Table S2). Comparison to matched unstimulated control cells at 8 and 24 h time points revealed an increase in the number of significantly upregulated genes in stimulated cells over time, while the number of downregulated genes remained similar (Fig. 1a). Stimulation with inactivated *Leptospira* and the bivalent vaccine induced 752 and 918 differentially expressed genes (DEGs) common to these stimuli, representing 53% and 56% of all DEGs, at 8 and 24 h, respectively (Fig. 1b). This indicates a strong response, common to stimulation with both inactivated *Leptospira* strains and a non-adjuvanted *Leptospira* vaccine by the 030-D cell line (Fig. 1b). In addition, a heat map of all DEGs showed similar expression changes in magnitude and directionality across both *Leptospira* strains and the bivalent vaccine (Fig. 1c). Stimulation with the excipient did not induce significant changes on the mRNA level when compared to the unstimulated control, suggesting that the cellular responses observed in 030-D cells are solely induced by *Leptospira* (Fig. 1b,c). The expression patterns of the inactivated *Leptospira* or the bivalent *Leptospira* vaccine specific DEGs (Fig. 1b) largely followed the same trend (Fig. 1c). For further analyses, we focused on the 752 and 918 DEGs common to stimulation with either *Leptospira* strains and the non-adjuvanted *Leptospira* vaccine at 8 and 24 h as these were highly expressed and therefore considered suitable as vaccine potency biomarkers.

**Figure 2:**
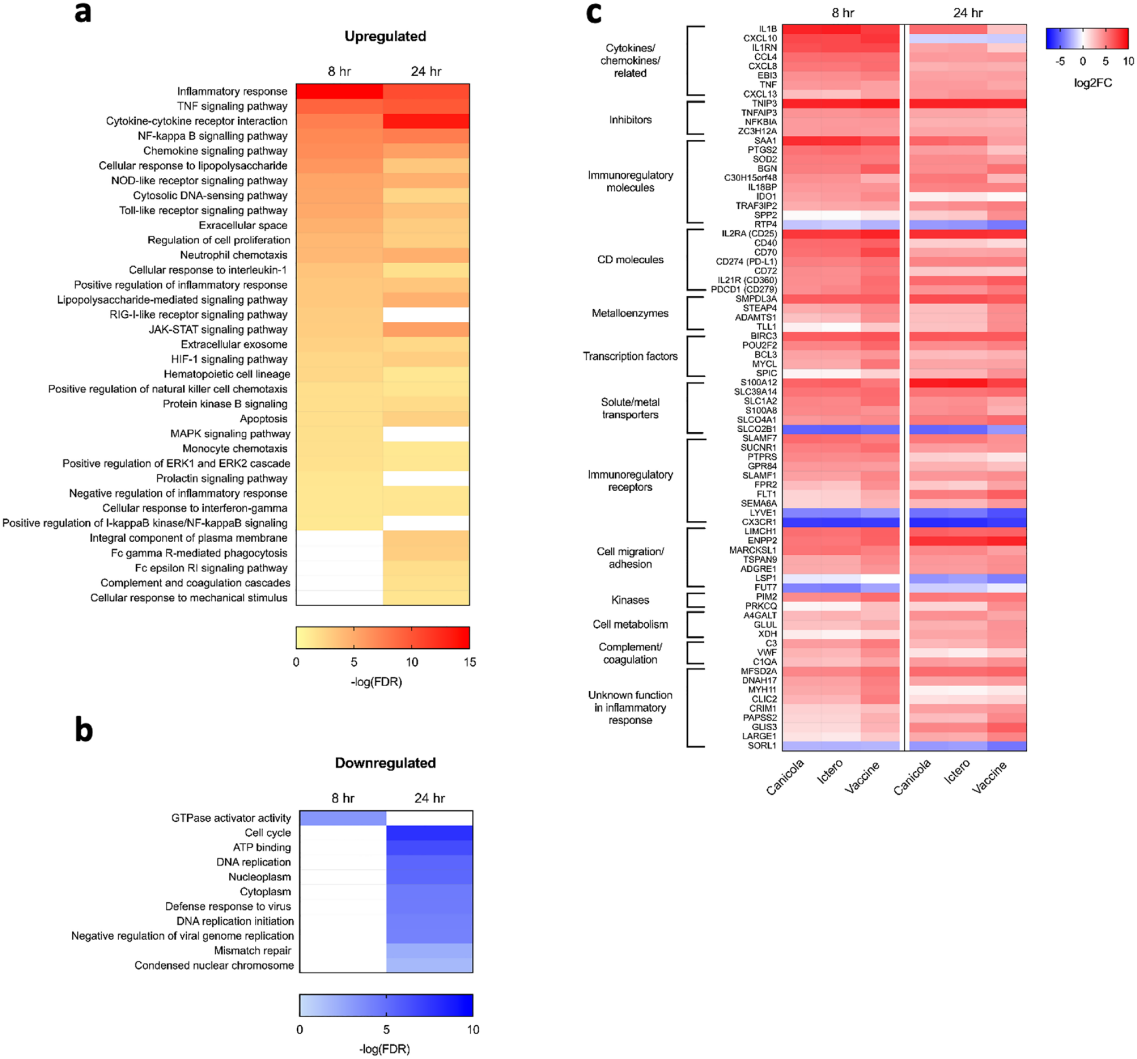
Common responses to the inactivated *L. interrogans* serogroup Canicola or Icterohaemorrhagiae and bivalent *Leptospira* vaccine B after 8 and 24 h stimulation. Pathways enriched for (**a**) upregulated and (**b**) downregulated genes found in common to stimulation with the inactivated *L. interrogans* serogroup Canicola or Icterohaemorrhagiae and bivalent *Leptospira* vaccine containing the same *Leptospira* strains from manufacturer B shown in Fig. 1b (FC ≥ 2; P < 0.001, FDR 5%). 752 and 918 common DEGs found at 8 and 24 h stimulation were included in pathway analysis. Blank spaces indicate non-significant or undetected pathways. (**c**) Among 752 and 918 DEGs that were found in common to all three stimuli at 8 and 24 h stimulation, respectively, 78 DEGs reached FC ≥ 16 16 in at least one of the conditions compared to the unstimulated control and were therefore considered as highly expressed. Heatmap shows these highly expressed genes grouped by function. The color key represents the changes from most downregulated (dark blue) to most upregulated genes (red). Each row is a gene, and each column is a stimulus (average of n = 3 replicates).

The upregulated DEGs common to inactivated *L. interrogans* serogroups Canicola and Icterohaemorrhagiae, and the bivalent vaccine B clustered within several expected categories associated with inflammation and innate immunity, pointing towards a strong innate immune response (P < 0.001, FDR 5%, FC ≥ 2; Fig. 2a). In contrast, the downregulated DEGs common to the *Leptospira* strains and the bivalent vaccine B were mainly associated with pathways involved in cellular processes at the 24 h time point (Fig. 2b). We further analysed 78 DEGs that were found significantly expressed by the stimulation with both *Leptospira* strains and the bivalent *Leptospira* vaccine B (FC ≥ 2), and strongly differentially expressed (FC ≥ 16) after stimulation with at least one of these stimuli (Fig. 2c) as stronger responses should be easier to detect in an in vitro assay. Among the strongest induced genes identified at the 8 h time point, were genes encoding the cytokines IL-1β and TNF-α, and the chemokines CXCL-10, CCL-4, and CXCL-8, suggesting stimulation with inactivated *Leptospira* strains and the bivalent vaccine B induces a strong pro-inflammatory response in the canine 030-D cell line. Other strongly upregulated genes at the 8 h time point known to be involved in the innate immune response included genes encoding the acute phase protein SAA1, the enzymes SOD2 and IDO1, costimulatory molecule CD40, metalloreductase STEAP4, calcium- and zinc-binding protein S100A8, succinate receptor SUCNR1, MARCKSL1, C3 and VWF. Besides genes involved in the pro-inflammatory response, several genes that inhibit this process were upregulated at 8 h including the gene encoding anti-inflammatory IL-1 receptor antagonist IL1RN, and genes involved in the termination of the NF-κB response, such as TNIP3, TNFAIP3 and NFKBIA. Furthermore, the upregulation of most of the genes encoding pro-inflammatory cytokines and chemokines was time-dependent and had decreased at the 24 h time point. Among genes that showed higher expression at the 24 h time point compared to 8 h were those encoding chemokine CXCL-13, calcium-, zinc- and copper-binding protein S100A12, vascular endothelial growth factor receptor FLT1 and IL-18 binding protein IL18BP (Fig. 2c).

### Proteome analysis of canine 030-D cells stimulated with inactivated *Leptospira *and bivalent *Leptospira* vaccine B

**Figure 3:**
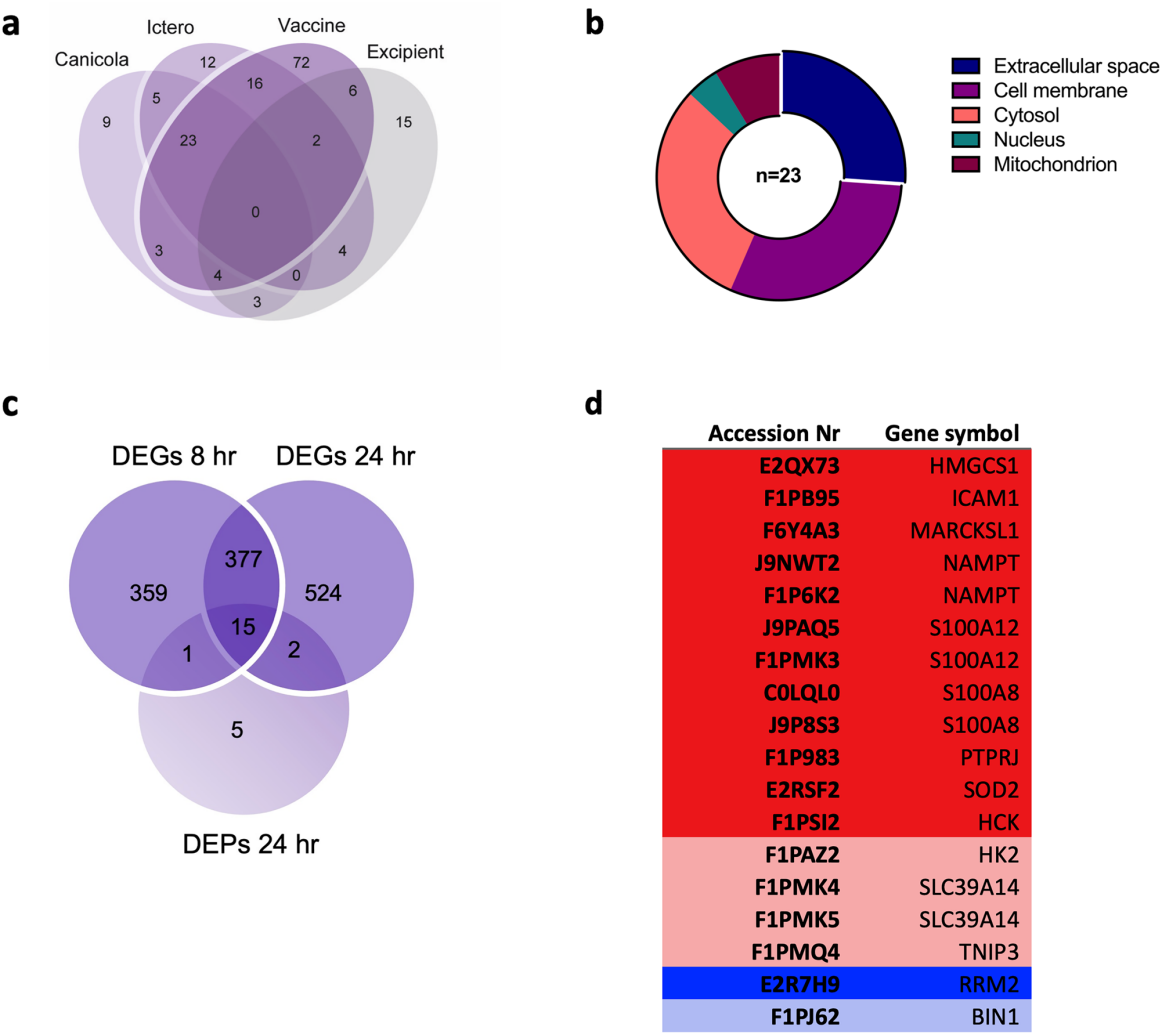
Analysis of the proteome of 030-D cells stimulated with the inactivated *L. interrogans* serogroup Canicola or Icterohaemorrhagiae or bivalent *Leptospira* vaccine B. (**a**) Venn diagram of DEPs found after 24 h stimulation with the inactivated *L. interrogans* serogroup Canicola or Icterohaemorrhagiae, bivalent *Leptospira* vaccine, or vaccine excipient as non-antigen control (all manufacturer B). Proteins were considered quantitative DEPs if found in at least two of the triplicates, showed at least twofold change in expression compared to unstimulated control (FC ≥ 2) and reached statistical significance (P < 0.01). Additionally, proteins were considered qualitative DEPs if found 3 × in the stimulated sample and absent in the unstimulated sample or the other way round. (**b**) Overview of the subcellular localization of 23 DEPs that were found after stimulation with the inactivated *Leptospira* strains and bivalent vaccine. (**c**) Venn diagram showing that 18 of the 23 DEPs found after 24 h stimulation with both *Leptospira* strains and the vaccine were also found on the transcriptome level at 8 and 24 h. (**d**) These 18 DEPs common to all three stimuli correspond to 14 unique genes. Upregulated DEPs are shown in red and downregulated DEPs in blue. DEPs found with quantitative analysis are indicated with bright colors, while DEPs found with qualitative analysis are indicated with dim colors.

Next, we performed unbiased profiling of protein expression changes in lysates of 030-D cells stimulated with inactivated *L. interrogans* serogroup Canicola or Icterohaemorrhagiae, bivalent *Leptospira* vaccine B containing these two *Leptospira* strains, and vaccine excipient from company B using LC–MS/MS (Fig. 3). A total of 2,623 proteins were identified (Supplementary Table S3). Proteins that were detected in at least two of the three replicates, met statistical significance (p < 0.01) and showed at least twofold change in expression (FC ≥ 2) compared to the unstimulated control were considered quantitative DEPs. In addition, DEPs that were either found three times in the stimulated condition and not in the unstimulated control cells or were three times detected in the unstimulated control cells and not in the stimulated conditions were considered qualitative DEPs. Compared to the unstimulated control cells at each time point, the expression changes of quantitative DEPs were more robust after 24 h stimulation than at 8 h (Supplementary Fig. S1), therefore we analysed DEPs at 24 h time point. At 24 h, 55 proteins were quantitative, and 125 proteins were qualitative DEPs. Because some DEPs were detected both by quantitative and qualitative analysis, this resulted in 174 DEPs. The overlap of DEPs found in 030-D cells stimulated with both *Leptospira* strains, the bivalent *Leptospira* vaccine B and the excipient is shown in a Venn diagram (Fig. 3a). Although specific DEPs were induced by each of the stimuli, these were mostly identified qualitatively and downregulated. Because no statistical analysis can be applied to qualitative DEPs, we considered these proteins to be less suitable as potential biomarkers for *Leptospira* vaccine potency. In contrast, most DEPs that were common to the stimulation with inactivated *Leptospira* strains and bivalent *Leptospira* vaccine B were identified quantitatively. Of 23 common DEPs, 17 were upregulated and 6 were downregulated, and all showed similar change in expression for all three stimuli (Supplementary Fig. S1 and Supplementary Table S3). Next, common DEPs were annotated for subcellular localization using gene ontology terms for cellular compartments. Most DEPs associated with the cytosol, cell membrane or extracellular space (Fig. 3b). Out of 23 common DEPs, 18 were also found on the transcriptome level as DEGs (Fig. 3c). These 18 molecules that were identified both on the transcriptome and proteome level corresponded to 14 unique genes (Fig. 3d) of which 6 (MARCKSL1, S100A12, S100A8, SLC39A14, SOD2 and TNIP3) were highly expressed (FC > 16; Fig. 2c). Interestingly, none of the cytokines and chemokines that are required for the propagation of the immune response were detected in the lysate of stimulated cells, suggesting that these molecules were not present intracellularly and potentially already secreted at this time point or undetected by mass spectrometry because of low protein abundance (Fig. 3).

### Highly similar responses induced by two different bivalent *Leptospira* vaccines

**Figure 4:**
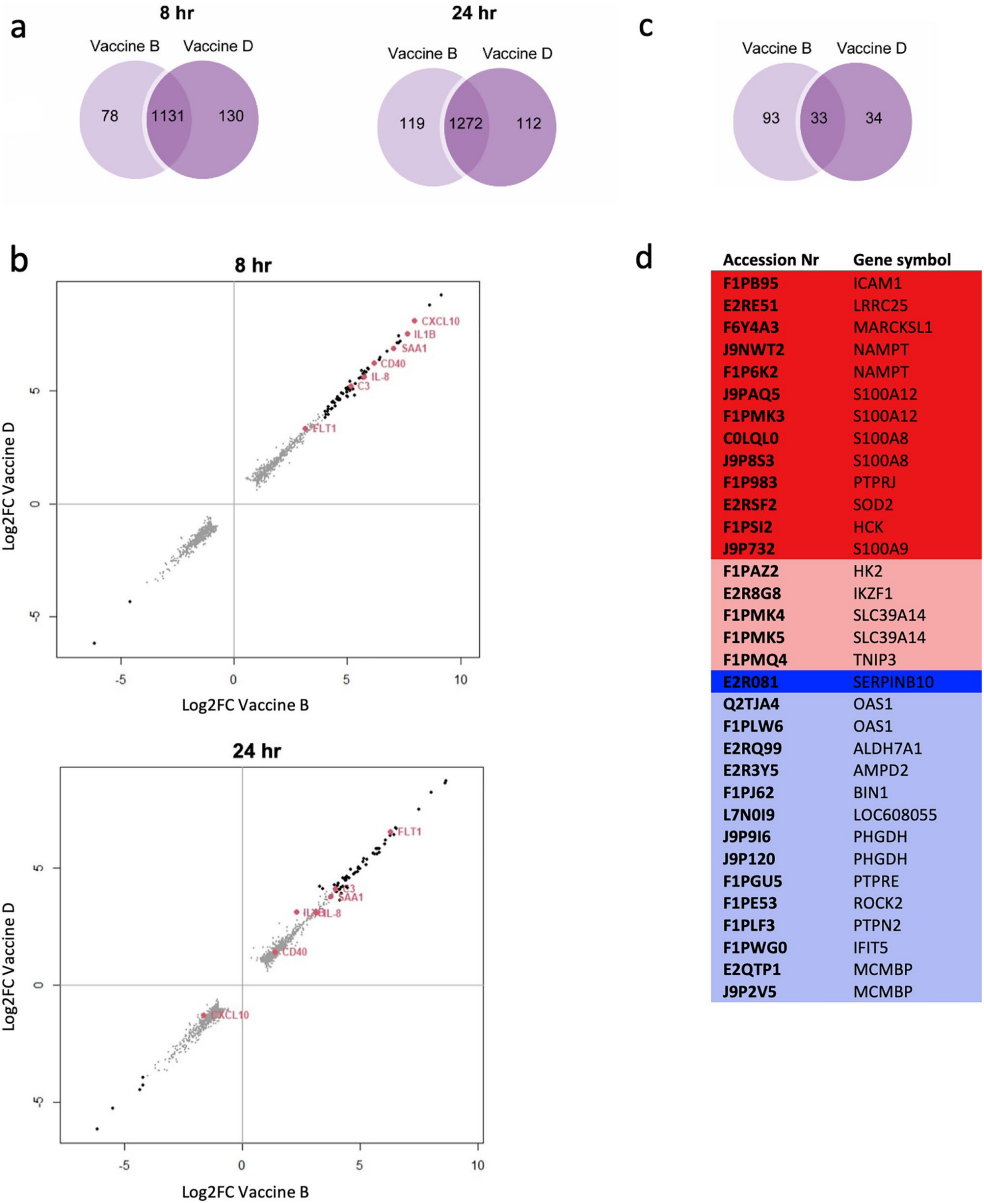
Comparison of DEGs and DEPs induced by bivalent *Leptospira* vaccines B and D. (**a**) Venn diagram of DEGs (FC ≥ 2; P < 0.001, FDR 5%) found after 8 and 24 h stimulation with bivalent *Leptospira* vaccines from two manufacturers (coded B and D) containing the inactivated *L. interrogans* serogroup Canicola or Icterohaemorrhagiae. (**b**) DEGs found after 8 and 24 h stimulation with bivalent vaccines B and D. DEGs with FC ≥ 2 are indicated in grey, while highly expressed DEGs with FC ≥ 16 are indicated in black. Additionally, some of the highly expressed DEGs are highlighted in red with the gene symbol next to it. (**c**) Venn diagram of DEPs found after 24 h stimulation with bivalent *Leptospira* vaccines B and D. (**d**) 33 DEPs induced by both vaccines correspond to 26 unique genes. Upregulated DEPs are shown in red and downregulated DEPs in blue. DEPs found with quantitative analysis are indicated with bright colors, while DEPs found with qualitative analysis are indicated with dim colors.

Because potency testing is an important part of the quality control process for a final vaccine product, we next compared changes in the transcriptome and proteome of 030-D cells induced by bivalent canine *Leptospira* vaccines from manufacturers B and D (Fig. 4). Both vaccines contain the inactivated *L. interrogans* serogroup Canicola and Icterohaemorrhagiae strains and are non-adjuvanted. The number of significantly upregulated and downregulated genes increased over time in 030-D cells stimulated with bivalent *Leptospira* vaccine B and D (Supplementary Fig. S2). Interestingly, 1131 and 1272 common DEGs, representing more than 80% of the total DEGs, were found in 030-D cells stimulated with both vaccines for 8 and 24 h, respectively (Fig. 4a). This suggests that it should be possible to identify potential targets that could be used for development of a biomarker-based in vitro assay for assessment of *Leptospira* vaccine potency. Next, we compared the expression of DEGs found in common in 030-D cells stimulated with bivalent vaccine B or vaccine D (Fig. 4b). Stimulation with both vaccines induced similar changes in gene expression for all DEGs in common (shown in grey). In addition, the expression of highly induced DEGs (FC > 16; shown in black) such as genes encoding IL-1β, IL-8, CXCL-10, SAA1, CD40, FLT-1, and C3 (shown red) was similar at 8 and 24 h time-points for both vaccines (Fig. 4b). The expression of the genes induced in 030-D by the two vaccines aligned with the DEGs induced by the *Leptospira* strains (Fig. 2c). Furthermore, DEPs specific for each of the vaccines were detected, although these were mostly downregulated and identified qualitatively (Fig. 4c). Both *Leptospira* vaccines induced expression of 33 common DEPs (Fig. 4c) of which 18 were upregulated and 15 were downregulated (Fig. 4d). The 33 DEPs that were in common to stimulation with vaccine B and D corresponded to 26 genes (Fig. 4d). The upregulated common DEPs aligned with DEPs induced by the two inactivated *Leptospira* strains (Fig. 3d).

### Secreted molecules as potential biomarkers of leptospiral vaccine potency

**Figure 5:**
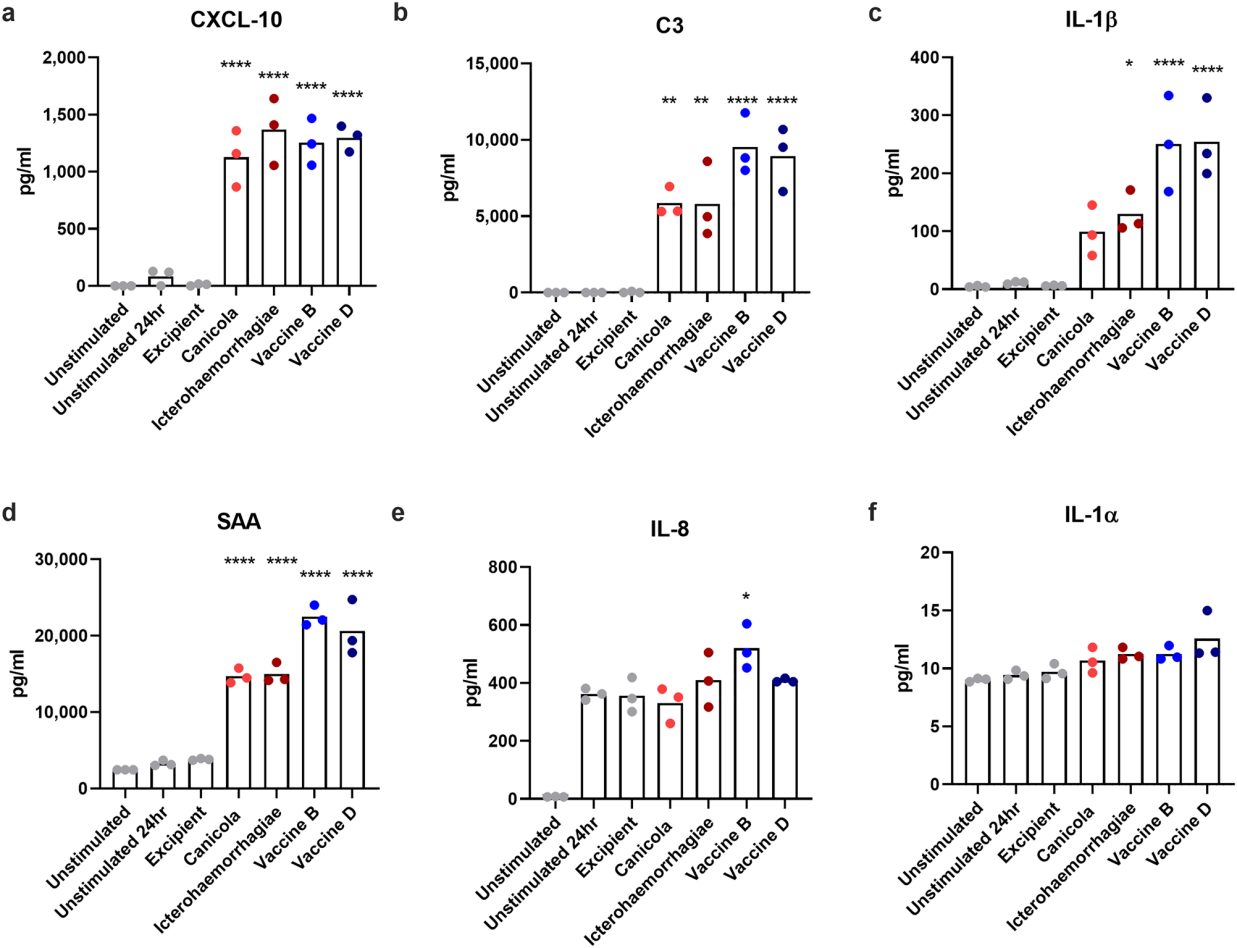
Expression of proteins associated with innate immune responses in the supernatant of stimulated 030-D cells. 030-D cells were stimulated for 24 h with whole-inactivated *L. interrogans* serogroup Canicola, Icterohaemorrhagiae, bivalent vaccine B, its excipient, or bivalent vaccine D. The supernatants were assayed for the presence of (**a**) CXCL-10, (**b**) C3, (**c**) IL-1β, (**d**) SAA, (**e**) IL-8 and (**f**) IL-1α. Stimulations were performed in triplicate. Dots represent values of each replicate and bars represent the mean. Three independent experiments were performed with similar outcome. A representative experiment is shown. A one-way ANOVA combined with Dunnett’s multiple comparisons test was used to test for significance. *p < 0.05, **p < 0.01, ****p < 0.0001 compared to the unstimulated control 24 h.

Because molecules involved in immune cell signalling such as cytokines and chemokines are secreted and were not found in the proteomics dataset, we verified some of the targets that were identified in the transcriptome dataset by ELISA or MagPix. Based on the availability of dog-specific monoclonal antibodies, we selected canine CXCL-10, IL-8, C3, SAA, S100A12, IL-1α and IL-1β and assessed their presence in the supernatants of 030-D cells stimulated for 24 h with the inactivated *L. interrogans* serogroup Canicola or Icterohaemorrhagiae, vaccine excipient, bivalent *Leptospira* vaccine (all company B), or bivalent *Leptospira* vaccine (company D). The CXCL-10, C3 and SAA amounts significantly increased after stimulation with both *Leptospira* strains and bivalent *Leptospira* vaccines compared to the unstimulated control at 24 h (Fig. 5a,b,d). The IL-1β amount significantly increased after stimulation with serogroup Icterohaemorrhagiae and both vaccines compared to the unstimulated control at 24 h. An increase in IL-1β secretion was found in Canicola stimulated cells as well, however, this was not statistically significant (Fig. 5c). Stimulation with the excipient did not induce secretion of CXCL-10, C3, IL-1β and SAA proteins, suggesting the upregulation of these proteins was *Leptospira* specific (Fig. 5). In addition, only vaccine B significantly upregulated IL-8 compared to the unstimulated control at 24 h. However, due to the high IL-8 level in both excipient stimulated and unstimulated control cells at 24 h of culture, measurement of IL-8 protein expression appears unsuitable for assessing *Leptospira* vaccine potency in vitro (Fig. 5e). Low IL-1α amounts were measured in all samples (Fig. 5f). In contrast, S100A12 was found upregulated in all conditions except in unstimulated control cells at 0 h (Supplementary Table S4). While changes in protein expression of CXCL-10, C3, IL-1β and SAA after stimulation with *Leptospira* and vaccines aligned well with the gene expression dataset, this was not the case for IL-8, IL-1α and S100A12 (Figs. 2c, 5 and Supplementary Table S4).

## Discussion

We analysed responses of the canine 030-D cell line induced by inactivated *Leptospira* and bivalent *Leptospira* vaccines by RNA sequencing and LC–MS/MS. This study aimed to gain insight in canine innate immune responses to *Leptospira* vaccines and to identify potential biomarkers indicative of *Leptospira* vaccine potency for future development of in vitro biomarker-based assays to assess *Leptospira* vaccine quality*.* To achieve this, the 030-D cell line was first stimulated with a non-adjuvanted canine *Leptospira* vaccine containing inactivated *L. interrogans* serogroups Canicola and Icterohaemorrhagiae, and the respective inactivated *Leptospira* strains present in this vaccine (all company B). Additionally, 030-D cells were stimulated with a bivalent, non-adjuvanted *Leptospira* vaccine from company D, containing the same serogroups, to investigate whether similar responses are induced by different *Leptospira* vaccines. The analysis of the transcriptome and proteome of stimulated cells resulted in a list of DEGs and DEPs. Related to these, the protein expression of some of the targets identified was assessed in the supernatant of stimulated 030-D cells using ELISA or MagPix assays considered as most suitable to use for future assessment of vaccine potency in vitro.

To verify whether the responses of the 030-D cell line used resembled that of innate immune cells, our transcriptome dataset was compared to transcriptome signatures of 17 immune cell types identified by Monaco et al. in the human PBMC pool^37^ (Supplementary Table S1). The ABIS RNA-seq tool relies on the detection of a specific signal pattern in a heterologous transcriptome dataset based on the expression of selected genes specific for an immune cell type^37^. This analysis showed highest similarity of 030-D cell line to non-conventional and intermediate monocytes (Supplementary Table S1). This outcome is in line with previous studies that used the 030-D cell line to study innate immune responses in dog^31,32^. Although a high score for the naïve CD4 T cells was found, the expression of conventional T cell markers or any of the genes associated with the T cell receptor was not detected. Based on the signature matrices provided by ABIS tool, we hypothesize that the high score for naïve CD4 T cells is probably related to genes which are highly expressed both in monocytes and naïve CD4 T cells^37^. However, it should be noted that this analysis lacks macrophage specific signatures and was developed specifically for human PBMC. Furthermore, the dog genome is less well annotated compared to that of humans, therefore the outcome of this analysis should be interpreted as an indication for best cell type match, rather than evidence-based characterisation.

Overall, the analysis of the 030-D transcriptome showed that the two inactivated *Leptospira* strains and a bivalent vaccine containing the same *Leptospira* serovars (all from company B) induce a strong innate immune response (Fig. 2). DEGs found in 030-D cells stimulated with individual *Leptospira* strains and vaccine clustered with several pathways associated with the activation of the innate immune response (Fig. 2a). Stimulation of 030-D cells with the inactivated *Leptospira* and bivalent vaccine resulted in 752 and 918 DEGs with similar levels of expression after 8 and 24 h, respectively (Fig. 1). A significant upregulation of mRNA expression of pro-inflammatory cytokines IL-1β and TNF-α, and chemokines CXCL-10, CCL-4 and CXCL-8 which are necessary for the propagation of the immune response was found after stimulation with *Leptospira* strains and the vaccine (FC > 16; Fig. 2c). Furthermore, albeit lower in expression, a significant increase in IL-6, CCL-3 and CCL-5 mRNA expression was found after stimulation with all three stimuli as well (Supplementary Table S2). These results are in line with a recent study that showed elevated IL-6, IL-8, TNF-α and CCL-3 mRNA levels in canine whole blood after stimulation with *Leptospira*^42^. The same study showed that IL-1β mRNA was only upregulated after stimulation with a non-pathogenic *L. biflexa* serovar Patoc strain, while a pathogenic *L. interrogans* serovar Copenhageni strain downregulated the IL-1β mRNA expression^42^. Differences between our and the previously published study might be due to different *Leptospira* strains or tools for detection of gene expression used. Nevertheless, the upregulation of IL-1β, IL-6 and TNF-α mRNA expression suggests a strong innate response of 030-D cells induced by the inactivated *Leptospira* strains and *Leptospira* vaccine used in this study. Immune responses to naturally occurring *Leptospira* infections have been mainly described in humans. Significantly higher concentrations of IL-1β, IL-2, IL-4, IL-6, IL-8, IL-10, IL-17A, and TNF-α were found in sera of leptospirosis patients^43,44^. Another study reported elevated protein levels of IL-1β, IL-6 and TNF-α in sera of leptospirosis patients and experimentally infected mice^45^, and elevated protein levels of IL-1, IL-8 and TNF-α were found in canine whole blood after stimulation with *Leptospira* bacteria^42^, suggesting live and inactivated *Leptospira* induce similar responses in different species.

Pathogens express a variety of PAMPs, which can activate several PRRs simultaneously and induce a diverse innate immune response necessary for subsequent shaping of an effective adaptive immune response to a specific pathogen. Therefore, some of the highest scoring immune pathways identified in Fig. 2a could be involved in the production of the pro-inflammatory cytokines IL-1β, TNF-α and IL-6, and chemokines IL-8, CCL-5 and CXCL-10 found in this study (Supplementary Fig. S3). The activation of Toll-like receptors (TLRs) (Supplementary Fig. S3a) by leptospiral PAMPs has been previously described^46,47^. The cytokines produced in response to TLR activation may be used to activate the TNF (Supplementary Fig. S3b) and MAPK (Supplementary Fig. S3d) signalling pathways involved in the propagation of cell adhesion, migration, and proliferation. Besides TLR signalling, stimulation with *Leptospira* and the vaccines triggered both the C-type lectin receptor signalling (Supplementary Fig. S3c) and cytosolic DNA sensing pathways (Supplementary Fig. S3f). In addition, factors from the complement system and coagulation cascade are also involved in the first line of innate immune response to pathogens (Supplementary Fig. S3e). Activation of the complement system results in the opsonization of the pathogen by the complement C3b molecule, recruitment of macrophages and enhanced phagocytosis. The crosstalk between the complement receptors and PRRs modulates subsequent cytokine secretion and T cell differentiation upon activation.

Whereas many significant DEGs were identified upon stimulation with inactivated *Leptospira* and bivalent *Leptospira* vaccine (Figs. 1, 2), the number of significant DEPs found in the proteome of stimulated 030-D cells was rather low (Fig. 3). This is probably because not all genes are translated into proteins, and because most secreted proteins cannot be detected in the cell lysate as they were secreted before cell harvesting^48^. Furthermore, some DEPs that were detected both in the transcriptome and proteome were associated with the same gene symbol, further reducing the number of molecules that were detected both on the mRNA and protein level (Fig. 3d). Most likely, this redundancy in DEPs is caused by posttranslational modifications and alternative splicing, which were considered during LC–MS/MS data processing. Therefore, the list of highly expressed DEGs (Fig. 2c) appeared more suitable for selection of candidates for identification of potential biomarkers.

When comparing responses induced by two non-adjuvanted *Leptospira* vaccines containing inactivated *L. interrogans* serogroup Canicola and Icterohaemorrhagiae as antigens, we found an 80% overlap in DEGs with similar expression levels (Fig. 4). This finding suggests that an assay based on one or several common biomarkers could be developed for different canine *Leptospira* vaccines with similar antigen content. Vaccine induced responses on transcriptome and proteome were comparable to responses induced by individual *Leptospira* strains (Figs. 2, 3 and 4). However, more complex *Leptospira* vaccines (e.g., tetravalent adjuvanted vaccines) should be evaluated in the future to verify whether a set of potential biomarkers similar to those found in the present study is induced.

For identification of biomarkers that correlate with vaccine quality we focused on DEGs encoding secreted molecules that were common to stimulation with either *Leptospira* strains and non-adjuvanted *Leptospira* vaccines for several reasons. First, detection of secreted molecules, indicative of vaccine potency, with ELISA-based assays is an interesting approach for development of biomarker-based in vitro assays for quality control purposes. ELISA-based assays are cost-effective, easy to perform and could be standardized between vaccine manufacturers. Second, genes of many secreted molecules were among DEGs with FC > 16, allowing measuring the expression of these genes quantitatively in vitro. Third, molecules commonly induced by inactivated *Leptospira* strains and *Leptospira* vaccines could be used for quality control assessment of both the final vaccine and intermediate products by a standardized ELISA-based assay. Based on high gene expression after stimulation with *Leptospira* and *Leptospira* vaccines (FC > 16; Figs. 2c and 4b), and the availability of commercial canine specific antibodies, we assessed the expression of CXCL-10, IL-8, SAA, C3, IL-1α, IL-1β and S100A12 in the supernatant of stimulated 030-D cells. Although IL-1α mRNA was expressed after stimulation with *Leptospira* and the vaccines (Supplementary Table S2), protein expression of this molecules in the supernatant of stimulated 030-D cells was not *Leptospira* specific (Fig. 5f). The IL-8 and S100A12 mRNA were highly expressed in cells stimulated with *Leptospira* and vaccines (Fig. 2c) and high expression of S100A12 was found in the proteome of these cells as well (Figs. 3d, 4d). Although both IL-8 and S100A12 were measured in the supernatant of stimulated cells, high levels of these proteins were already detected in the supernatants of the unstimulated and excipient stimulated cells (Fig. 5e and Supplementary Table S4). Therefore, IL-1α, IL-8 and S100A12 are not suitable candidates for development of a *Leptospira* vaccine potency assay. CXCL-10, IL-1β, C3 and SAA were found among highly expressed DEGs (FC > 16). The IL-1β, C3 and SAA mRNA expression reduced at 24 h compared to 8 h stimulation, while the CXCL-10 mRNA expression completely shut down at 24 h compared to 8 h (Fig. 2c), suggesting a rapid induction and inhibition of these responses. Because IL-1β, CXCL-10, C3 and SAA are secreted molecules, they were not detected in the proteome analysis of the cell lysates (Figs. 3, 4d and Supplementary Fig. S1). However, IL-1β, CXCL-10, C3 and SAA were found significantly upregulated in the supernatant of 030-D cells stimulated with inactivated *Leptospira* strains or *Leptospira* vaccines (Fig. 5a–d). Because the expression of these four molecules was induced by *Leptospira* strains or *Leptospira* vaccines specifically, we consider them interesting candidates for development of a biomarker-based in vitro assay for assessment of canine *Leptospira* vaccine quality in the future.

Knowledge on the role of IL-1β, CXCL-10, C3 and SAA in *Leptospira* vaccine induced immunity is limited. IL-1β, the classical pro-inflammatory cytokine, mediates the expansion of antigen specific CD4 and CD8 T cells, their differentiation into effector cells, migration into periphery and development of a long-term memory response. Furthermore, immunization of mice in presence of IL-1β significantly improved the immunogenicity of subunit and inactivated vaccines^49,50^. We have shown that *Leptospira* vaccine strains induce IL-1β mRNA and protein expression partly via TLR4 in canine monocyte-derived dendritic cells^47^. Together, this suggests that IL-1β is an important cytokine in *Leptospira* vaccine induced immunity. CXCL-10 is a pro-inflammatory chemokine involved in the recruitment of T cells. CXCL-10 binds to CXCR3 expressed on immune cells including activated CD4^+^ Th1 lymphocytes. Upon activation, Th1 cells secrete cytokines such as IFN-γ, which in turn induce production of CXCL-10 by different immune cells in a positive feedback loop^51^. Previous studies associated CXCL-10 with vaccine-elicited immunity in humans^52^ and mice^53^, and an increase in CXCL-10 mRNA was recently described in hamsters immunized with a LPS-depleted *Leptospira* vaccine^54^. Together, this suggests that CXCL-10 might be an interesting marker for assessment of *Leptospira* vaccine quality. SAA has been described as an opsonin of pathogenic Gram-negative bacteria*,* thereby promoting phagocytosis^55^. Elevated SAA levels have been reported during leptospirosis in humans^56^. Our results suggest that SAA may be involved in innate immune responses to *Leptospira* vaccines in dogs as well. Previously, complement factor C3 has been shown to play a role in antigen delivery to B cells within lymph node follicles, thereby enhancing humoral immunity^57^. In mice, C3 has been shown to play a crucial role in inducing humoral and cellular responses to influenza vaccination^58^. Although pathogenic *Leptospira* evolved mechanisms that can cleave C3 to inhibit the complement system^59,60^, the upregulation of C3 by the inactivated *Leptospira* could be beneficial for the immune response elicited by inactivated canine *Leptospira* vaccines and underlines C3 as a promising marker for assessment of *Leptospira* vaccine potency.

In conclusion, this study identified several pro-inflammatory molecules involved in *Leptospira* vaccine-induced responses in canine monocytes. Specifically, IL-1β, CXCL-10, SAA and complement factor C3 may be candidate targets for the development of biomarker-based assays to test *Leptospira* vaccine potency in vitro. Other potential biomarkers indicative of vaccine quality were identified (Fig. 2c), but not assessed for presence in culture supernatants. Depending on future availability of antibodies, ELISA-based tests could be developed for QC purposes. Alternatively, expression could be measured by RT-qPCR. Future studies with non-compliant vaccines are needed to confirm whether these molecules are in fact suitable markers of *Leptospira* vaccine quality. Besides, studying the role of these molecules in innate immune responsiveness more in depth could give further insight in *Leptospira* pathogenicity and contribute to the understanding of the cellular mechanisms involved in the induction of immunity by inactivated *Leptospira* vaccines in the 030-D model or primary canine cells.

## Supplementary Information

Supplementary Information 1.

Supplementary Table 2.

Supplementary Table 3.

## Data Availability

The transcriptomics datasets generated during the current study are available in the GEO repository, (www.ncbi.nlm.nih.gov/geo/) under accession number GSE192945. The proteomics datasets generated are available in the PRIDE repository, [http://www.ebi.ac.uk/pride/archive/projects/PXD031875].
