# Supplementary material for: Transcriptome and proteome analysis of innate immune responses to inactivated Leptospira and bivalent Leptospira vaccines in canine 030-D cells
Source: Sci Rep. 2022 Aug 4;12:13418. doi: 10.1038/s41598-022-16457-z (PMC9352656; doi:10.1038/s41598-022-16457-z)
Supplement: Supplementary file 1 — Supplementary Information 1. [file 41598_2022_16457_MOESM1_ESM.pdf]

Transcriptome and proteome analysis of innate immune responses to inactivated *Leptospira* and bivalent *Leptospira* vaccines in canine 030-D cells

Andreja Novak[1,2], Jeroen L.A. Pennings[3], Larissa van der Maas[2], Hugo D. Meiring[2], Irene Ludwig[1], Saertje Verkoeijen[4], Victor Rutten[1,5], Femke Broere[1,6], Arjen Sloots[2]*

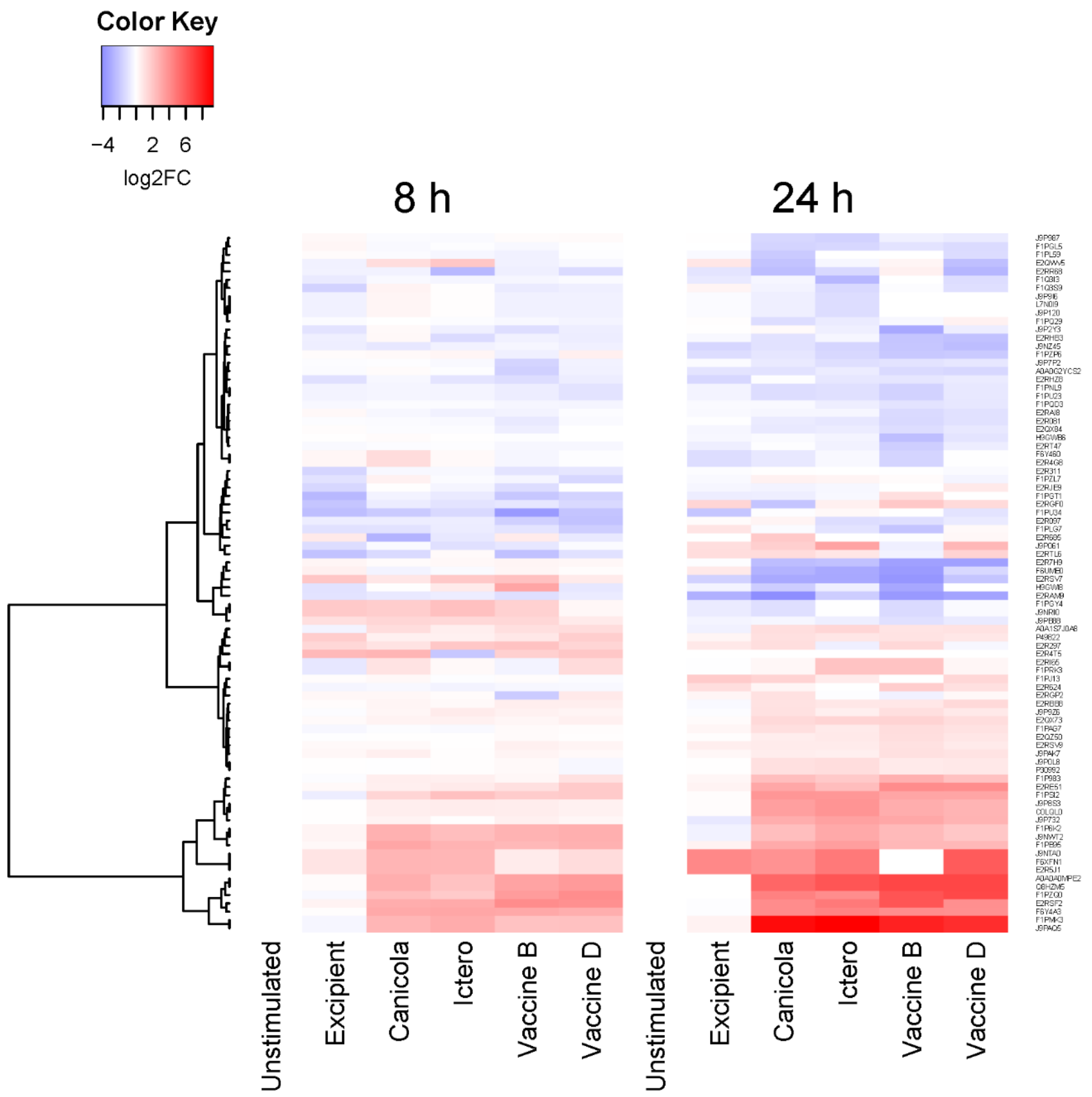

Figure S1. Heatmap of quantitative DEPs identified in 030-D cells. Shown are 36 quantitative DEPs found at 8 h together with 55 quantitative DEPs that were found at 24 h stimulation with the inactivated *L. interrogans* serogroup Canicola or Icterohaemorrhagiae, a bivalent *Leptospira* vaccine from manufacturer B (vaccine B), a bivalent *Leptospira* vaccine from manufacturer D (vaccine D), or vaccine excipient (84 quantitative DEPs in total). Proteins were considered quantitative DEPs if found in at least two of the triplicates, showed at least 2-fold change in expression (FC ≥ 2) and reached statistical significance ($P < 0.01$). The color key represents the changes (log2 scale), from most downregulated (dark blue) to most upregulated genes (red). Each row is a UniProt ID, and each column is a stimulus (average of n=3 replicates).

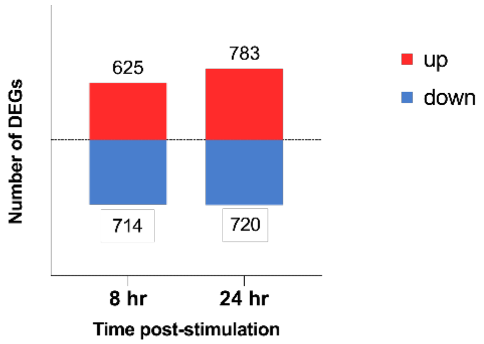

Figure S2. DEGs (FC ≥ 2; P < 0.001, FDR 5%) in canine 030-D cells stimulated with bivalent vaccine B and D. The number (y-axis) and directionality (upregulated=positive y-space, downregulated=negative y-space) of DEGs after 8 and 24 hr stimulation with bivalent *Leptospira* vaccines from different manufacturers (coded B and D).

a

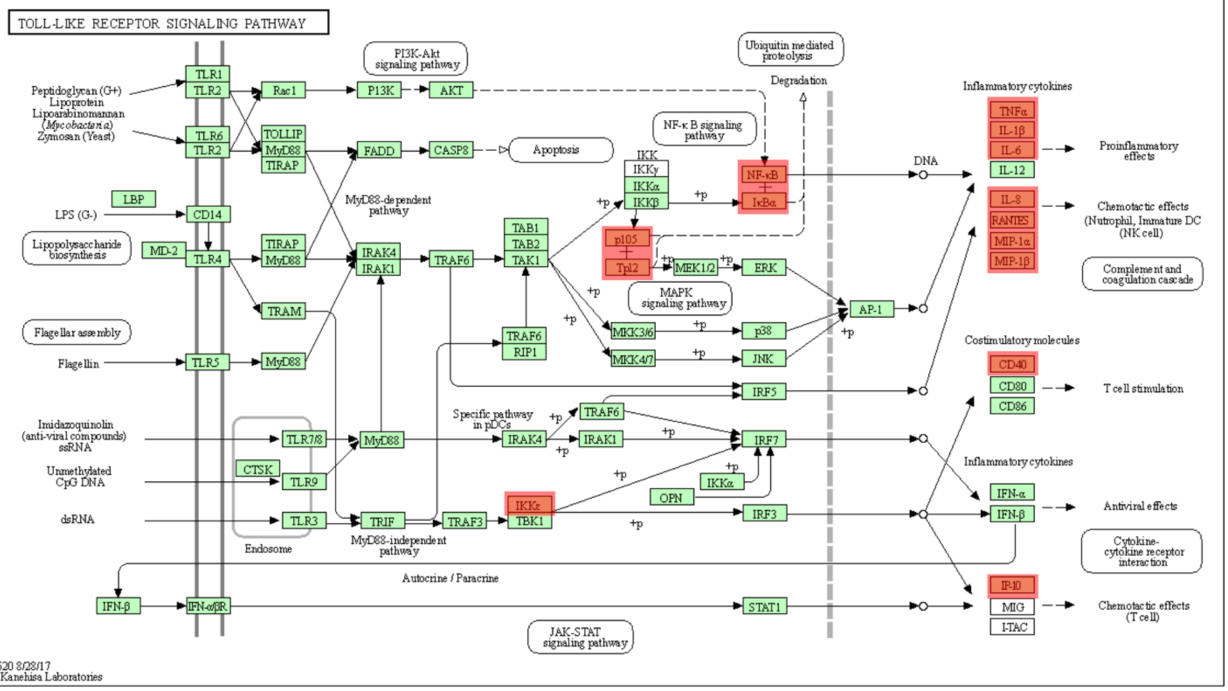

TOLL-LIKE RECEPTOR SIGNALING PATHWAY
Peptidoglycan (G+)
Lipoprotein
Lipoarabinomannan
(Mycobacteria)
Zymosan (Yeast)
LBP
LPS (G-)
Lipopolysaccharide biosynthesis
MD-2
Flagellar assembly
Flagellin
Imidazoquinolin
(anti-viral compounds)
ssRNA
Unmethylated CpG DNA
dsRNA
TLR1
TLR2
TLR6
TLR2
CD14
TLR4
TLR5
TLR7/8
CTSK
TLR9
TLR3
Endosome
IFN-β
IFN-α/βR
Rac1
PI3K
AKT
PI3K-Akt signaling pathway
TOLLIP
MyD88
TIRAP
FADD
CASP8
Apoptosis
MyD88-dependent pathway
TIRAP
MyD88
IRAK4
IRAK1
TRAM
MyD88
TRAF6
TAB1
TAB2
TAK1
TRAF6
RIP1
MyD88
Specific pathway in pDCs
IRAK4
IRAK1
TRAF6
TRIF
TRAF3
MyD88-independent pathway
IKKε
TBK1
OPN
IKKα
Autocrine / Paracrine
STAT1
JAK-STAT signaling pathway
IKK
IKKγ
IKKα
IKKβ
NF-κB signaling pathway
p105
Tpl2
MEK1/2
ERK
MAPK signaling pathway
MKK3/6
MKK4/7
p38
JNK
IRF5
IRF7
IRF3
Ubiquitin mediated proteolysis
Degradation
NF-κB
IκBα
AP-1
DNA
Inflammatory cytokines
TNFα
IL-1β
IL-6
IL-12
Proinflammatory effects
IL-8
RANTES
MIP-1α
MIP-1β
Chemotactic effects
(Nutrophil, Immature DC (NK cell)
Complement and coagulation cascade
Costimulatory molecules
CD40
CD80
CD86
T cell stimulation
Inflammatory cytokines
IFN-α
IFN-β
Antiviral effects
Cytokine-cytokine receptor interaction
IP-10
MIG
I-TAC
Chemotactic effects
(T cell)
+p
+u
04620 8/28/17
(c) Kanehisa Laboratories

b

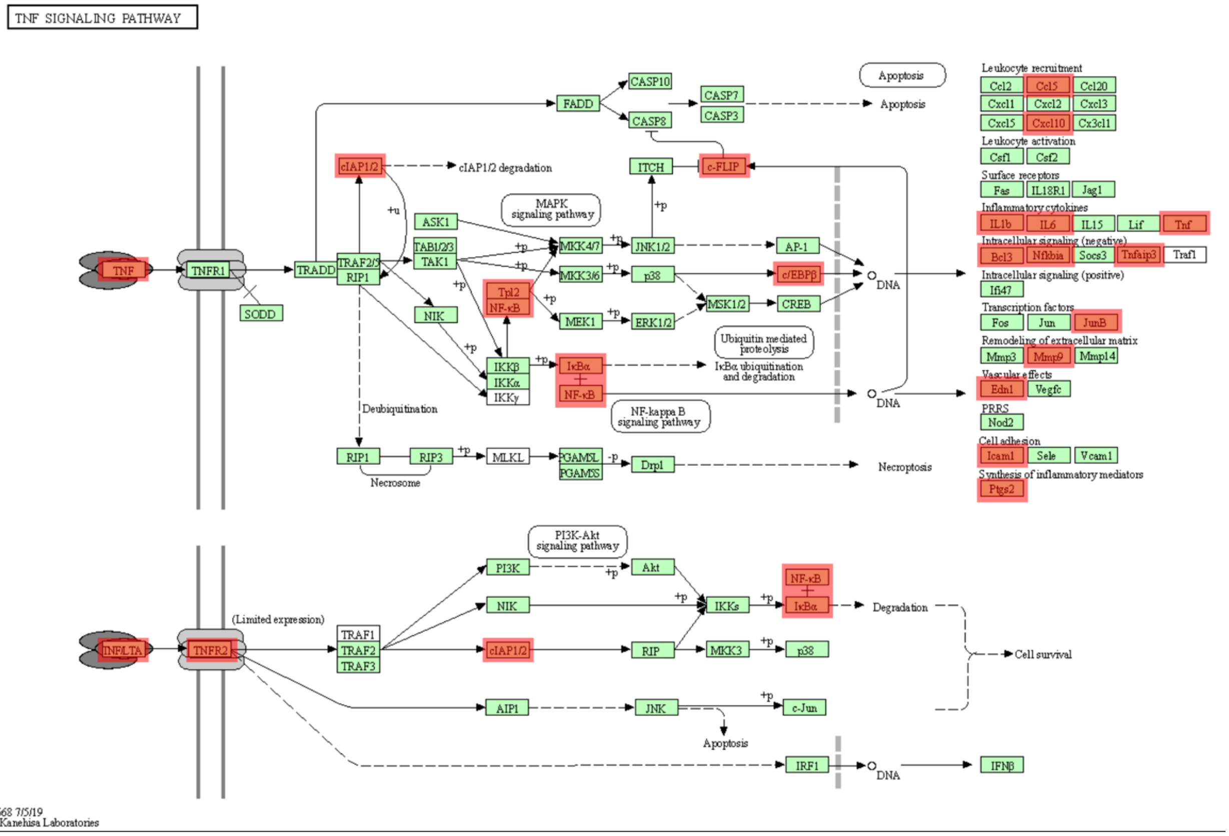

TNF SIGNALING PATHWAY
TNF
TNFR1
SODD
TRADD
TRAF2/5
RIP1
cIAP1/2
cIAP1/2 degradation
FADD
CASP10
CASP8
CASP7
CASP3
Apoptosis
Apoptosis
ITCH
c-FLIP
ASK1
TAB1/2/3
TAK1
MAPK signaling pathway
MKK4/7
MKK3/6
JNK1/2
p38
AP-1
c/EBPβ
Tpl2
NF-κB
MEK1
ERK1/2
MSK1/2
CREB
NIK
IKKβ
IKKα
IKKγ
IκBα
NF-κB
Ubiquitin mediated proteolysis
IκBα ubiquitination and degradation
NF-kappa B signaling pathway
DNA
DNA
Deubiquitination
RIP1
RIP3
MLKL
PGAM5L
PGAM5S
Drp1
Necrosome
Necroptosis
Leukocyte recruitment
Ccl2
Ccl5
Ccl20
Cxcl1
Cxcl2
Cxcl3
Cxcl5
Cxcl10
Cx3cl1
Leukocyte activation
Csf1
Csf2
Surface receptors
Fas
IL18R1
Jag1
Inflammatory cytokines
IL1b
IL6
IL15
Lif
Tnf
Intracellular signaling (negative)
Bcl3
Nfkbia
Socs3
Tnfaip3
Traf1
Intracellular signaling (positive)
Ifi47
Transcription factors
Fos
Jun
JunB
Remodeling of extracellular matrix
Mmp3
Mmp9
Mmp14
Vascular effects
Edn1
Vegfc
PRRS
Nod2
Cell adhesion
Icam1
Sele
Vcam1
Synthesis of inflammatory mediators
Ptgs2
PI3K-Akt signaling pathway
PI3K
Akt
NIK
IKKs
NF-κB
IκBα
Degradation
Cell survival
TNF/LTA
TNFR2
(Limited expression)
TRAF1
TRAF2
TRAF3
cIAP1/2
RIP
MKK3
p38
AIP1
JNK
c-Jun
Apoptosis
IRF1
DNA
IFNβ
+p
+u
-p
04668 7/5/19
(c) Kanehisa Laboratories

c

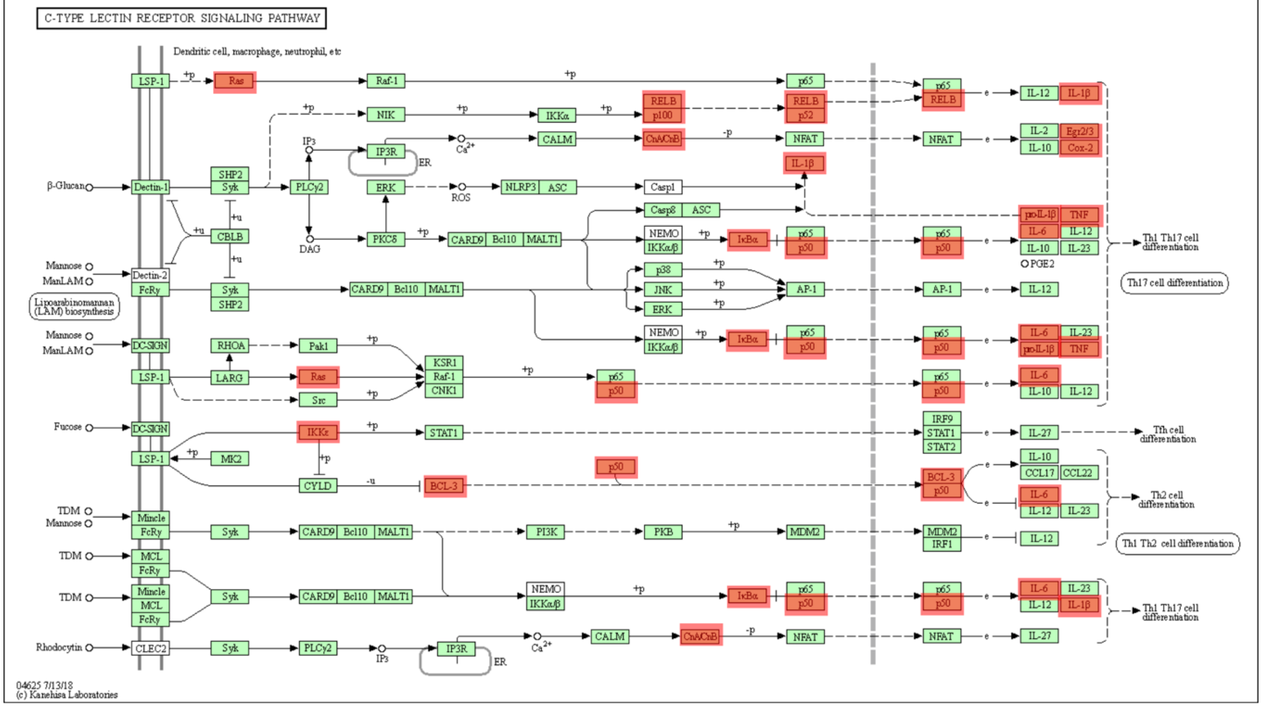
C-TYPE LECTIN RECEPTOR SIGNALING PATHWAY
Dendritic cell, macrophage, neutrophil, etc
LSP-1
Ras
Raf-1
p65
RELB
p100
p52
NIK
IKKα
IP3
IP3R
ER
Ca2+
CALM
CN(A/CnB)
NFAT
IL-1β
β-Glucan
Dectin-1
SHP2
Syk
PLCγ2
ERK
ROS
NLRP3
ASC
Casp1
Casp8
CBLB
DAG
PKCδ
CARD9
Bcl10
MALT1
NEMO
IKKα/β
IκBα
p50
p38
JNK
AP-1
Mannose
ManLAM
Dectin-2
FcRγ
Lipoarabinomannan (LAM) biosynthesis
DC-SIGN
RHOA
Pak1
KSR1
CNK1
LARG
Src
Fucose
IKKε
STAT1
MK2
CYLD
BCL-3
IRF9
TDM
Mincle
PI3K
PKB
MDM2
IRF1
MCL
CLEC2
Rhodocytin
IL-12
IL-2
Egr2/3
IL-10
Cox-2
pro-IL-1β
TNF
IL-6
IL-23
PGE2
IL-27
CCL17
CCL22
Th1 Th17 cell differentiation
Th17 cell differentiation
Tfh cell differentiation
Th2 cell differentiation
Th1 Th2 cell differentiation
04625 7/13/18
(c) Kanehisa Laboratories

d

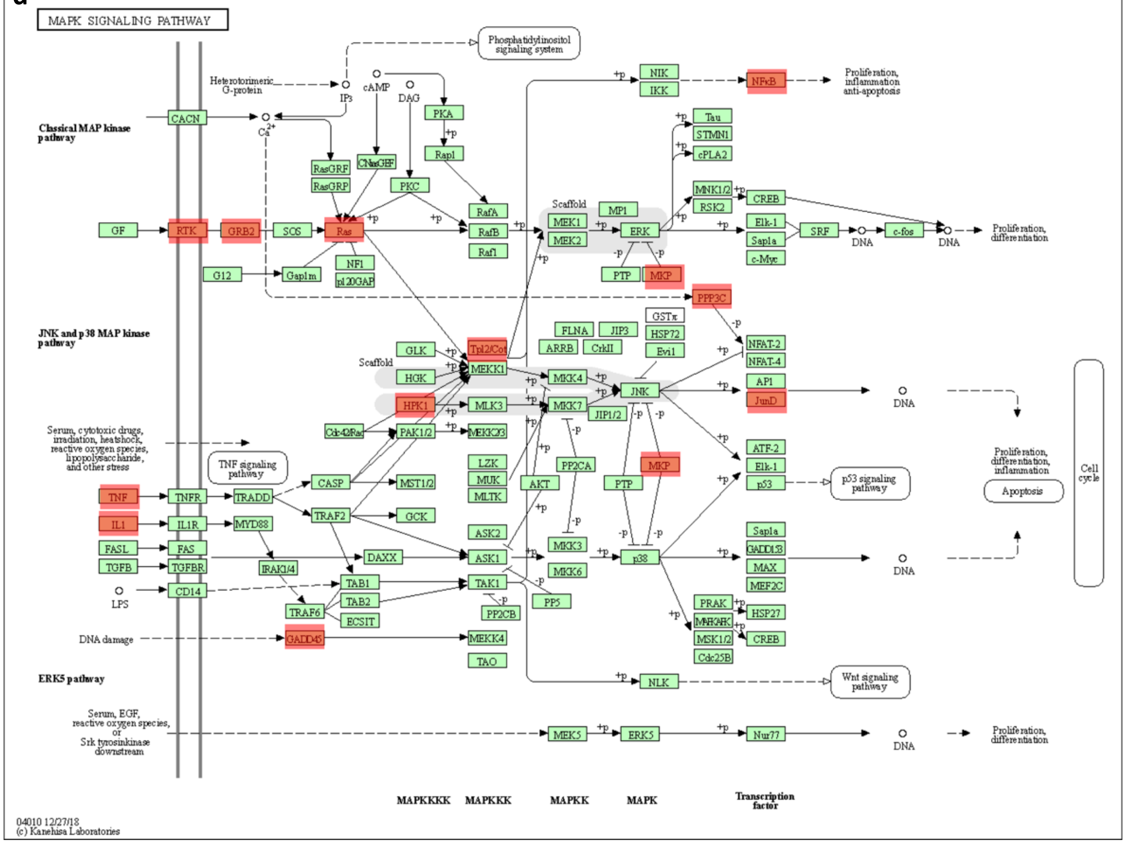
MAPK SIGNALING PATHWAY
Classical MAP kinase pathway
JNK and p38 MAP kinase pathway
ERK5 pathway
Phosphatidylinositol signaling system
Heterotrimeric G-protein
IP3
cAMP
DAG
CACN
Ca2+
PKA
Rap1
RasGRF
RasGRP
CNrasGEF
PKC
GF
RTK
GRB2
SOS
Ras
NF1
p120GAP
G12
Gap1m
RafA
RafB
Raf1
Scaffold
MP1
MEK1
MEK2
ERK
PTP
MKP
PPP3C
NIK
IKK
NFκB
Proliferation, inflammation, anti-apoptosis
Tau
STMN1
cPLA2
MNK1/2
RSK2
CREB
Elk-1
Sap1a
c-Myc
SRF
c-fos
DNA
Proliferation, differentiation
FLNA
JIP3
ARRB
CrkII
GSTπ
HSP72
Evi1
NFAT-2
NFAT-4
AP1
JunD
GLK
HGK
HPK1
Tpl2/Cot
MEKK1
MLK3
MEKK2/3
Cdc42/Rac
PAK1/2
MKK4
MKK7
JNK
JIP1/2
Serum, cytotoxic drugs, irradiation, heatshock, reactive oxygen species, lipopolysaccharide, and other stress
TNF signaling pathway
TNF
TNFR
TRADD
CASP
MST1/2
LZK
MUK
MLTK
AKT
PP2CA
ATF-2
p53
p53 signaling pathway
Proliferation, differentiation, inflammation
Apoptosis
Cell cycle
IL1
IL1R
MYD88
TRAF2
GCK
ASK2
ASK1
MKK3
MKK6
p38
FASL
FAS
DAXX
TGFB
TGFBR
IRAK1/4
TAB1
TAB2
TAK1
PP5
LPS
CD14
TRAF6
ECSIT
PP2CB
MAX
MEF2C
PRAK
MAPKAPK
HSP27
MSK1/2
Cdc25B
DNA damage
GADD45
MEKK4
TAO
NLK
Wnt signaling pathway
Serum, EGF, reactive oxygen species, or Src tyrosinkinase downstream
MEK5
ERK5
Nur77
MAPKKK
MAPKK
MAPK
Transcription factor
04010 12/27/18
(c) Kanehisa Laboratories

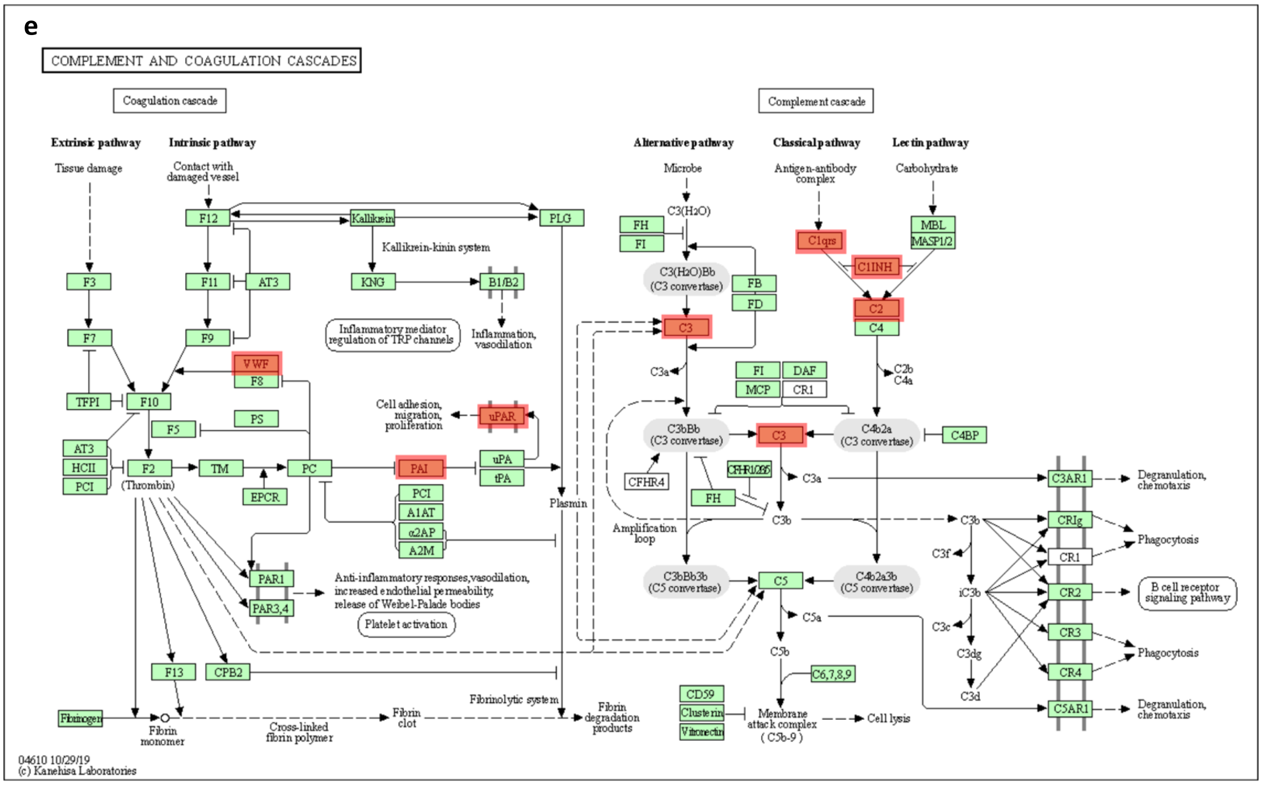

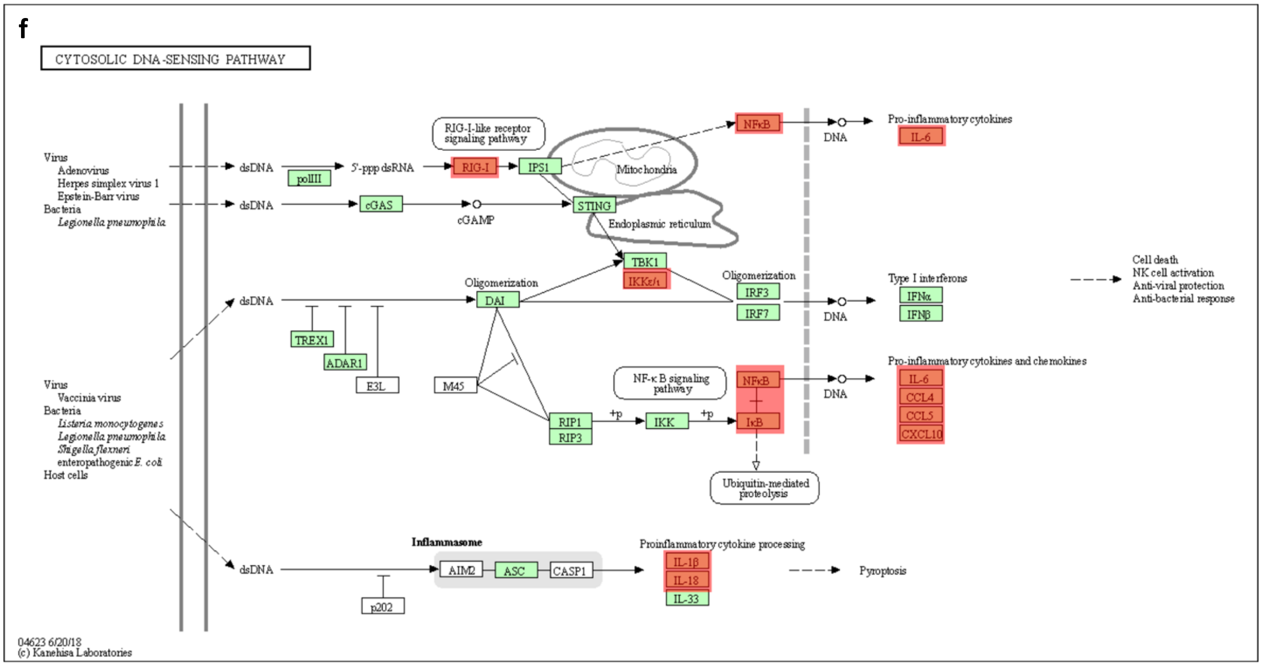

Figure S3. Upregulated pathways in canine 030-D cells stimulated with the inactivated *L. interrogans* serogroup Canicola or Icterohaemorrhagiae, or bivalent *Leptospira* vaccine B. Shown are **a)** Toll-like receptor **b)** TNF **c)** C-type lectin receptor and **d)** MAPK signaling pathways, **e)** complement and coagulation cascades, and **f)** cytosolic DNA sensing pathway. Upregulated DEGs (FC ≥ 2; P < 0.001, FDR 5%) found in stimulated cells at 8 h are highlighted in red. cfa04620 (a), cfa04668 (b), cfa04625 (c), cfa04010 (d), cfa04610 (e), cfa04623 (f) pathway maps were obtained with permission from https://www.kegg.jp/kegg/kegg1.html.

Table S1. Transcriptomic immune profile of 030-D cells

Cell type	Average%	SD%
**Monocytes NC+I**	6.74	2.06
**Monocytes C**	0.26	0.56
**pDCs**	0.25	0.03
**mDCs**	-0.01	0.03
**Neutrophils LD**	0.67	0.48
**Basophils LD**	0.07	0.01
**NK**	-0.30	0.13
**T CD4 Naive**	3.82	0.59
**T CD4 Memory**	-1.63	0.36
**T CD8 Naive**	-1.81	0.42
**T CD8 Memory**	0.78	0.20
**MAIT**	-0.15	0.06
**T gd Vd2**	0.52	0.18
**T gd non-Vd2**	0.04	0.01
**B Naive**	-0.02	0.05
**B Memory**	0.00	0.05
**Plasmablasts**	0.15	0.02

Gene expression profiles of the 030-D gene expression dataset were deconvoluted to those of 17 human cell types found in PBMC using the ABIS RNA-seq tool described by Monaco *et al.*[35]. The values represent the average gene expression score and standard deviation (SD) for the 39 samples analyzed in this study. NC, non-classical; I, intermediate; C, classical, pDC, plasmacytoid dendritic cells; mDC, myeloid dendritic cells; LD, low-density.

Table S4. The concentration of S100A12 protein in the supernatant of 030-D cells stimulated for 24 h with whole inactivated *L. interrogans* serogroup Canicola, Icterohaemorrhagiae, bivalent *Leptospira* vaccine B, its excipient, or bivalent *Leptospira* vaccine D.

Condition	Absorbance
**Unstimulated 0 h**	<0.115
**Unstimulated 24 h**	>2.19
**Excipient**	>2.19
**Canicola**	>2.19
**Ictero**	>2.19
**Vaccine B**	>2.19
**Vaccine D**	>2.19

The supernatants were assayed for the presence of S100A12. Stimulations were performed in triplicate. Three independent experiments were performed with similar outcome. A representative experiment is shown. Values of 0.115 and 2.19 were the means of the lowest and the highest absorbance measured in the S100A12 standard curve, respectively. The S100A12 values in all samples were above the highest concentration of the standard curve except the unstimulated 0 h.
